# Attention-deficit/hyperactivity disorder in children with overactive bladder; a case-control study

**DOI:** 10.15171/jrip.2016.41

**Published:** 2016-08-09

**Authors:** Parsa Yousefichaijan, Mojtaba Sharafkhah, Mohammad Rafiei, Bahman Salehi

**Affiliations:** ^1^Department of Pediatrics, Arak University of Medical Sciences, Arak, Iran; ^2^Students Research Committee, Arak University of Medical Sciences, Arak, Iran; ^3^Department of Biostatistics and Epidemiology, Arak University of Medical Sciences, Arak, Iran; ^4^Department of Psychiatry, School of Medicine, Arak University of Medical Sciences, Arak, Iran

**Keywords:** Attention deficit/hyperactivity disorder, Urinary bladder, Overactive

## Abstract

**Introduction:** Attention-deficit/hyperactivity disorder (ADHD) is the most common childhood psychiatric disorder. This disorder is more prevalent in some chronic diseases.

**Objectives:** To investigate ADHD in children with overactive bladder.

**Patients and Methods:** A number of 92 children with overactive bladder and 92 healthy children without overactive bladder (age range of both groups 5 to 12 years old) were included in this study as case and control groups, respectively. Participants were selected from children who had referred to a pediatric clinic in Arak city, Iran. ADHD types (inattentive, hyperactive-impulsive, and mixed) were diagnosed by Conner’s Parent Rating Scale and Diagnostic and Statistical Manual of Mental Disorders IV-TR (DSM-IV-TR) criteria. Data were analyzed by chi-square and t tests.

**Results:** In both groups, 51 children (27.7%) had ADHD. The prevalence of ADHD in the case group (33 cases, 35.9%) was significantly higher than the control group (18 cases, 19.6%) (*P* = 0.021). Inattentive ADHD was observed in 22 participants (23.9%) of the case group and nine participants of the control group (9.7%) (*P* = 0.047). Despite this significant difference, three (3.2%) and four (4.3%) children were affected by hyperactive-impulsive ADHD (*P* = 0.73), and eight (8.6%) and five (5.4%) children were affected by mixed ADHD (*P* = 0.42) in the case and control groups, respectively.

**Conclusion:** ADHD bladder is significantly more common in children with overactive bladder than healthy children. The observed correlation between ADHD and overactive bladder makes psychological counseling mandatory in children with overactive bladder.

**Implication for health policy/practice/research/medical education:**

Attention-deficit/hyperactivity disorder (ADHD) is more prevalent in some chronic diseases. Aside from the relationship between nocturnal enuresis and ADHD, there may be an association between overactive bladder and ADHD. Hence, this study investigated the prevalence of ADHD in children with overactive bladder.

## Introduction

Attention deficit /hyperactivity disorder (ADHD) is the most common psychiatric disorder of childhood. According to the fourth edition of Diagnostic and Statistical Manual of Mental Disorders (DSM-IV) criteria, it has three types: inattentive, hyperactive-impulsive, and mixed (1).

ADHD affects 3% to 5% of all children in the United States (2). The causes of ADHD in children are not clearly known; however, evidence recognizes underlying genetic defect and central nervous system dysfunction as its main causes (3). Based on the studies, ADHD can be significantly associated with a variety of chronic diseases (4-6), depression (6), behavioral, emotional, language, and hearing disorders (7,8) and even illnesses such as epilepsy (5) and abnormal electroencephalogram in children (9).

As the evidence suggests, in addition to the aforementioned disorders, voiding dysfunction and bowel habit disorders can also be associated with ADHD in children (10-13). In this regard, Burgu and colleagues (11), McKeown et al (12), and Duel et al (13) showed that the prevalence of different types of voiding dysfunction and constipation are significantly higher in children with ADHD compared to healthy children. It was also found that enuresis can be related to the onset and worsening of symptoms in ADHD patients (14).

Children with an overactive bladder typically exhibit urinary frequency, urgency, and urge incontinence (15). The bladder in these children is functionally, but not anatomically, smaller than normal and exhibits strong uninhibited contractions. Approximately, 25% of children with nocturnal enuresis also have symptoms of an overactive bladder (15,16).

Although the etiology is poorly understood, disturbances in nerves, detrusor smooth muscle and urothelium are all thought to lead to overactive bladder. Overactive bladder is more likely to occur in association with certain risk factors (e.g. being female, increasing age) and a number of other medical conditions such as depression and anxiety (15). Some authors believe that, overactive bladder is a manifestation of a centrally located dysfunction that affects multiple systems, notably bowels, bladder, sexual and ejaculatory function, control of blood pressure, and even mood and behavior(17). Considering the possible relationship of overactive bladder and psychiatric disorders, its probable link with ADHD should be kept in mind.

## Objectives

Aside from the relationship between nocturnal enuresis and ADHD (11,18-20), there may be an association between overactive bladder and ADHD. Hence, this study investigated the prevalence of ADHD in children with overactive bladder.

## Patients and Methods

This case-control study was conducted on 184 five to twelve years old children (95 [51.63%] boys and 97 [52.71%] girls) who were referred to a pediatric clinic in Arak city, Iran. They were investigated in two groups based on the inclusion/exclusion criteria; 92 children with overactive bladder as the case group and 92 healthy children without overactive bladder as the control group.

Overactive bladder is a type of voiding dysfunction in children. In children with voiding dysfunction signs, kidney and urinary tract ultrasound with or without voiding cystourethrogram is recommended in order to diagnose the type of voiding dysfunction and examine neurogenic bladder and anatomical abnormalities of the bladder (16). Thus, in this study children with voiding dysfunction (at least for six months) underwent ultrasound and voiding cystourethrogram. Then according to clinical presentations, imaging, and rejection of other causes of voiding dysfunction, overactive bladder was diagnosed. Finally, only the children with overactive bladder (and no other types of voiding dysfunction) were included in the study as the case group.

Overactive bladder was defined as the presence of urinary frequency, urgency, and urge incontinence in medical history and the presence of dilation of the urethra with distal urethral narrowing and contraction of the bladder neck in voiding cystourethrogram (15,16).

Clinical interviews were carried out with the children and their parents to study the inclusion/exclusion criteria. The inclusion criteria were; 1) Being five to twelve years old; 2) Having overactive bladder according to its diagnostic criteria. The exclusion criteria were; 1) Having a history of considerable psychiatric disorders, mental retardation or nervous system disorders, 2) Having congenital and chromosomal abnormalities, 3) Having a history of considerable or chronic medical disorders such as seizure, asthma, diabetes, immune deficiency and malignancy, 4) Having a history of sleep apnea or other sleeping disorders which can cause ADHD-like symptoms, 5) Having a strong family history (first-degree relatives) of major psychiatric disorders. All participants and their parents signed an informed consent before participating in this study.

Mental retardation was defined as the intelligence quotient of 70 or less (19). The healthy participating children were selected from children who had referred to two hospitals for common cold and abdominal pain, as an outpatient. Matching method was used for selecting the healthy children and children were matched in both groups regarding age and gender. Demographic, clinical and perinatal data such as age, sex, birth weight, mother’s age at birth, gestational age, maternal education, household incomes, marital status of the parents, siblings, type of delivery and history of jaundice at birth were recorded.

ADHD was diagnosed according to DSM-IV criteria and the 48-item Conner›s Parent Rating Scale (CPRS-48). Based on DSM-IV criteria, ADHD has three types: inattentive, hyperactive-impulsive, and mixed (3,7,21,22). Inattentive ADHD was defined as the presence of six (or more) symptoms of inattention with fewer than six symptoms of hyperactivity-impulsivity for at least six months. Hyperactive-impulsive ADHD was defined as presence of six (or more) symptoms of hyperactivity-impulsivity with fewer than six symptoms of inattention for at least six months. Mixed ADHD was defined as presence of six (or more) symptoms of inattention and six (or more) symptoms of hyperactivity-impulsivity for at least six months (7).

CPRS was standardized by Conners and colleagues in 1999. It has two versions; 93-item and 48-item. In this research, the 48-item version has been used. This version of Conner’s questionnaire evaluates five factors of conduct, psychosomatic-impulsivity, hyperactivity, anxiety and learning problems scored from 0 (never) to 3 (very high). The score of each article is converted into t scores with the average of 50 and standard deviation of 10. If the t scores are two standard deviations higher than the average, the individual has a problem (23).

After CPRS-48 was completed by the parents and different types of ADHD were identified in the children, they were referred to an expert psychiatrist as the project administrator for the confirmation of ADHD diagnosis by clinical interview based on DSM-IV diagnostic criteria (3,7,21,22). Also, ADHD diagnosis was confirmed by evaluating its differential diagnosis such as hyperthyroidism (by measuring thyroid-stimulating hormone [TSH] and free T4 [FT4]) and lead poisoning (blood lead level >5-10 μg/dl) (25). Unlike doing thyroid tests, measuring blood lead level is not a routine procedure in examining the children suspected of having ADHD. However, clinical signs of lead poisoning and blood lead level were used to reach a more reliable diagnosis of ADHD and to roll out symptomatic and asymptomatic cases of lead poisoning (26).

### Ethical issues

The research followed the tenets of the Declaration of Helsinki; informed consent was obtained; and the research was approved by the ethical committee of Arak University of Medical Sciences.

### Statistical analysis

The collected data were analyzed with statistical package for the social sciences (SPSS) software version 18.0 (SPSS Inc., Chicago, USA). Descriptive statistics were used for frequency determination. Numerical data were expressed as mean±standard deviation (SD) and compared with *t* test. Categorical data were expressed as number (percentage) and compared with chi-square test. *P* values less than 0.05 were considered significant.

## Results

In this nine-month study (from December 2013 to September 2014), 124 children with overactive bladder were examined for having the inclusion and not having the exclusion criteria. Among them, 32 (24.2%) children were excluded and were replaced with other qualified participants. In order to find replacements, 36 other patients with overactive bladder were evaluated. Among the 32 excluded children, 24 (75%), 6 (18.7%) and 2 (6.2%) children were excluded due to their parents’ dissatisfaction to complete of CPRS-48 questionnaire, having a history of mental retardation and having a history of considerable psychiatric disorders (anxiety disorders), respectively. For the control group, 92 out of 108 investigated children were selected. The rest were excluded because of not completing the questionnaires.

The mean age and birth weight of all children were 8.12 ±1.69 years old and 2866.10±617.77 g, respectively. The mother’s age at birth was 25.32±5.64 years old (Table 1). Mother’s age at birth (*P*=0.017), maternal education (*P*=0.001), household incomes (*P*=0004) and type of delivery (*P*=0 .001) were significantly different in both groups. The average age of children with inattentive, hyperactive-impulsive and mixed ADHD were 8.8±1.81, 8.37±1.59 and 8.2±1.23 years old, respectively, which were not significant (*P*>0.05). Of the 184 participating children in both groups, 51 (27.7%) children had ADHD, among whom 31 (60.7%), 7 (13.7%) and 13 (25.4%) children were affected by inattentive, hyperactive-impulsive, and mixed types, respectively.

**Table 1:** Clinical and demographic characteristics in in the study groups

	**Case group (n=92)**	**Control group (n=92)**	**P value**
Age (mean ± SD) (year)	9.48±2.14	8.95±2.05	0.09
Gender (%)			0.5
Girl	47 (51.08)	50 (50)	
Boy	45 (53.2)	50 (50)	
Birth weight (mean ± SD) (g)	2788.26±614.91	2943.94±614.10	0.08
Birth weight^1^ (%)			0.82
ELBW	2 (2.2)	1 (1.1)	
VLBW	8 (8.7)	7 (7.6)	
LBW	16 (17.4)	13 (14.1)	
NLBW	66 (71.7)	71 (77.2)	
Gestational age (%)			0.017
Full term	63 (68.5)	79 (85.9)	
Premature (<37 wk)	25(27.2)	12(13)	
Post-term (>40 wk)	4 (4.3)	1 (1.1)	
Maternal education (%)			0.001
College	38 (41.3)	19 (20.7)	
High school	49 (53.3)	53 (57.6)	
Elementary school	5 (5.4)	20 (21.7)	
Mother's age at birth (mean ± SD) (year)	25.37±5.71	25.02±5.55	0.66
Household incomes^2^(%)			0.004
Low	55 (59.8)	47 (51.1)	
Middle	34 (37)	28 (30.4)	
High	3 (3.3)	17 (18.5)	
Marital status of the parents (%)			> 0.05
Intact marriage	76 (82.6)	75 (81.5)	
Separated	16 (17.4)	17 (18.5)	
Having siblings (%)	12 (13.04)	13 (14.1)	> 0.05
Type of mother’s delivery^3^ (%)‏			0.001
NVD	65 (70.6)	83 (90.2)	
CS	27 (29.3)	9 (9.7)	
ADHD types^4^ (%)	33 (35.9)	18 (19.6)	0.021
Newborn jaundice (%)	18 (19.5)	21 (22.8)	> 0.05

^1^ ELBW: extremely low birth weight (<1000 g), VLBW: very low birth weight (<1500 g), LBW: low birth weight (<2500 g), NLBW: normal birth weight (2500-4200 g). ^2^ Household incomes: low mean monthly incomes <5000000 Rials; moderate mean incomes between 5000000 and 10000000 Rials; high means incomes > 10000000 Rials. ^3^NVD: normal vaginal delivery, CS: caesarean section. ^4^ inattentive, hyperactive-impulsive, and mixed types.

**Figure 1:**
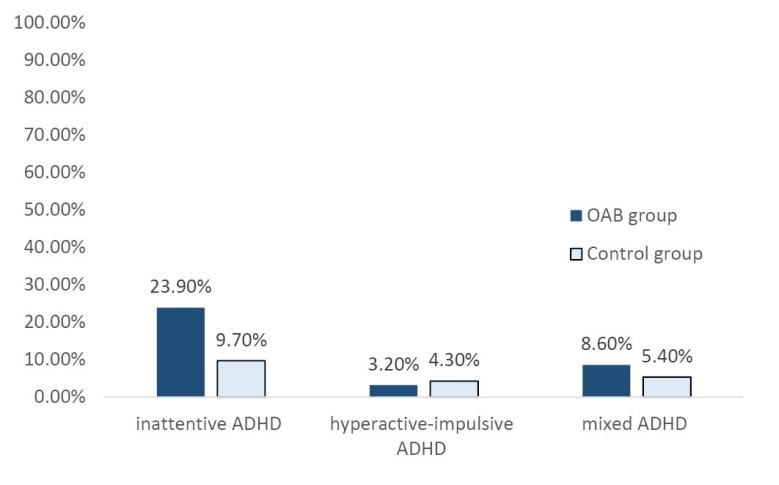
Frequency of ADHD types in the case and control groups.

ADHD with 33 cases (35.9%) in the overactive bladder group was significantly higher than the control group with 18 cases (19.6%) (*P* = 0.021). Although the frequency of inattentive ADHD in both groups (22 (23.9%) in the case group versus 9 (9.7%) in the control group; *P*=0.047) was significant, the difference of hyperactive-impulsive (3 (3.2%) versus 4 (4.3%); *P*=0.73) and mixed ADHD (8 [8.6%] versus 5 [5.4%]; *P*=0.42) was not significant in the two groups (Figure 1). There was a significant relationship between the birth weight (*P*=0.001), gestational age (*P*=0.001), marital status of the parents (*P*=0.001) and type of delivery (*P*=0.001) and ADHD types (Table 2).

**Table 2:** Clinical and demographic characteristics in the studied children with ADHD

	**Inattentive ADHD (n=31)**	**Hyperactive-impulsive ADHD (n=7)**	**Mixed ADHD (n=13)**	**P value**
Sex (%)				0.87
Girl	14 (45.1)	3 (42.8)	6 (46.1)	
Boy	17 (54.8)	4 (57.1)	7 (53.8)	
Birth weight (%)				0.001
ELBW	2 (6.4)	0 (0)	1 (7.6)	
VLBW	9 (29.03)	1 (14.2)	4 (30.7)	
LBW	10 (32.2)	5 (71.4)	5 (38.4)	
NLBW	10 (32.2)	1 (14.2)	3 (23.07)	
Gestational age (%)				0.001
Full term	6 (19.3)	4 (57.1)	7 (53.8)	
Premature (<37 wk)	23 (74.1)	3 (42.8)	6 (46.1)	
Post-term (>40 wk)	2 (6.4)	0 (0)	0 (0)	
Maternal education (%)				0.29
College	1 (3.2)	0 (0)	2 (15.3)	
High school	18 (58.06)	6 (85.7)	8 (61.5)	
Elementary school	12 (38.7)	1 (14.2)	3 (23.07)	
Household incomes(%)				0.44
Low	17 (54.8)	3 (42.8)	8 (61.5)	
Middle	13 (41.9)	4 (57.1)	4 (30.7)	
High	1 (3.2)	0 (0)	1 (7.6)	
Marital status of the parents (%)				0.001
Intact marriage	16 (51.6)	7 (100)	11 (84.6)	
Separated	15 (48.3)	0 (0)	2 (15.3)	
Having siblings (%)	2 (6.4)	0 (0)	1 (7.6)	>.05
Type of mother’s delivery (%)‏				0.001
NVD	13 (41.9)	2 (28.5)	5 (38.4)	
CS	18 (58.06)	5 (71.4)	8 (61.5)	
Newborn jaundice (%)	2 (6.4)	1 (14.2)	0 (0)	>.05

## Discussion

This study investigated the ADHD in children with overactive bladder. The frequency of ADHD in the overactive bladder group was significantly higher than the control group. However, unlike inattentive ADHD, the difference in frequency of hyperactive-impulsive and mixed ADHD was not significant in both groups.

To the authors’ knowledge, no study has been conducted on the relationship between ADHD and overactive bladder in children. However, different studies have been conducted on ADHD and other voiding dysfunctions in children. In 2003 Duel and colleagues (13) studied voiding dysfunction in 28 children with ADHD and 12 healthy children using dysfunctional voiding symptom survey. The total score of their survey was 19.0±5.3 for the ADHD group versus 5.83 for their control group, which was statistically significant (*P*=0.002). In a retrospective review of patients with ADHD, urinary incontinence and nocturnal enuresis, Crimmins and colleagues (27) showed that the presence of ADHD had a negative effect on the resolution of incontinence, with 68% of the ADHD patients becoming continent compared to 91% of the control group (*P*<0.01). Treatment noncompliance was found in 48% of the ADHD patients compared to 14% of control group (*P*<0.01).

In 2009 Elia and colleagues (28) investigated 344 children aged 6 to 12 years old. They reported that the enuresis group had a higher likelihood of inattentive symptoms than the non-enuretic group. In a study by Baeyens and colleagues (18), 86 children with enuresis were screened twice for the presence of ADHD in a two-year interval. Based on this study, the odds that a child with ADHD still has voiding problems after two years is 3.17 times higher than a child without ADHD. Burgu et al, studied voiding problems among 62 children with ADHD and 124 healthy children using the lower urinary tract symptom score.Mean±SD of their total lower urinary tract symptom score was 11.1±2.9 in ADHD patients with hyperactivity and 3.2±1.3 in the control group, which was statistically significantly (*P*<0.001) (11). Accordingly Shreeram et al (29) reported 9.89% prevalence for ADHD in children with nocturnal enuresis, and Yang et al (30) reported 28.3% prevalence of nocturnal enuresis in children with ADHD.

In this study, CPRS was the diagnostic tool for ADHD, and this diagnosis was confirmed in the next stage by DSM-IV-TR criteria. According to the evidence, this questionnaire and its different versions have good reliability and validity for the diagnosis of ADHD in children (31-35). The reliability and validity of CPRS were reported favorable by Gau and colleagues (31), Kumar et al (32). Al-Awad et al (33), Pal et al (34) and Rosenberg and colleagues (35) in both clinical and Chinese community children, Sudanese children, Bengali children, and India, respectively. Moreover, in a study by Abdekhodaie et al (24) in 2012, the sensitivity and specificity of the Persian version of CPRS for the diagnosis of ADHD in Iranian children were 90.3% and 81.2%, respectively.

According to earlier studies, ADHD onset is higher in boys than girls (7,36,37). However, based on our results although the incidence of all ADHD types were higher in the boys, this difference was not significant.

In some studies, the prevalence of ADHD in the general population of children in Iran was different from the overall frequency of ADHD types in healthy children of our study (19.6%). In the studies by Hebrani and colleagues (38) and Abdekhodaie et al (24) conducted in the northeast of Iran and Ghanizadeh et al (39) conducted in the south of Iran, also the studies by Meysamie et al (40) on three to six years old children, and also in the study by Azadbakht and colleagues (41) in Tehran, the frequencies were 12.3%, 12.3%, 10.1%, 17-25.8%, and 9.7%, respectively. According to these studies, it seems that the difference in the prevalence of ADHD between different studies and our study is because of geographical differences as well as different definitions of ADHD and its subtypes.

Considering (*a*) the significant relationship between ADHD and voiding dysfunction (especially nocturnal enuresis) (11,13,18,27-30), (*b*) the significant association between nocturnal enuresis and overactive bladder (16), and (*c*) the negative impact of ADHD on treatment response in children with nocturnal enuresis (18,27,37), the relationship between ADHD and overactive bladder is likely and important.

Although a significant association was found between ADHD and overactive bladder in our study, in clinical situation the relationship between these two disorders cannot be easily proved based on this result. Furthermore, we could not notice the comorbidity of ADHD cases because most of them were inattentive, not hyperactive-impulsive. Therefore, a patient with overactive bladder should be assessed with a psychological questionnaire or at least some questions be asked to reveal ADHD.

## Conclusion

ADHD is significantly more common in children with overactive bladder than healthy children. The observed correlation between ADHD and overactive bladder makes psychological counseling mandatory in children with overactive bladder. Since geographical differences seem to be effective in the prevalence of ADHD and no epidemiological study has been done on the prevalence of ADHD in the central part of Iran, further studies are needed. Also, further studies on the relationship between ADHD and overactive bladder are recommended for clinical application of such findings. Since assessing the severity of overactive bladder and its relationship with ADHD in children can be effective in showing the relationship between ADHD and overactive bladder, it is recommended to be considered in future studies.

## Limitations of the study

Among the limitations of our study, we may indicate lack of cooperation of some parents to fill in ADHD questionnaire and the psychiatric examination of the child performed by the psychiatrist. Although this criterion led to excluding some eligible children, we attempted to remove such limitation by encouraging the parents for possible usefulness of the study and help them fill in the questionnaire. In addition, lack of assessment the severity of overactive bladder and its relationship with ADHD, was another limitation of our study. Therefore, future studies with consideration of more clinical factors are recommended.

## Authors’ contribution

PY; participated in the design of the study, performed the data collection, performed the statistical analysis and served as the lead author of the manuscript. MS; participated in the design of the study, statistical analysis and in finalizing the manuscript. MR; participated in the design of the study and statistical analysis. BS; wrote some parts of the draft. All authors read and approved the final manuscript.

## Conflicts of interest

The authors declare no conflict of interest.

## Ethical considerations

Ethical issues (including plagiarism, data fabrication, double publication) have been completely observed by the authors.

## Funding/Support

This study was financially supported by Arak University of Medical Sciences (Grant # 878).
